# Improved African Swine Fever Detection for Environmental Samples in the Presence of Organic Contaminants

**DOI:** 10.1155/tbed/8841168

**Published:** 2024-12-31

**Authors:** Taeyong Kwon, Jordan T. Gebhardt, Eu Lim Lyoo, Natasha N. Gaudreault, Jessie D. Trujillo, Jason C. Woodworth, Chad B. Paulk, Cassandra K. Jones, Juergen A. Richt

**Affiliations:** ^1^Department of Diagnostic Medicine/Pathobiology, College of Veterinary Medicine, Kansas State University, Manhattan, Kansas 66506, USA; ^2^Department of Animal Sciences and Industry, College of Agriculture, Kansas State University, Manhattan, Kansas 66506, USA; ^3^Department of Grain Science and Industry, College of Agriculture, Kansas State University, Manhattan, Kansas 66506, USA

**Keywords:** African swine fever, ASFV, environmental sample, fomite, organic contaminants, surface

## Abstract

Geographical expansion and trans-continental transmission of the African swine fever virus (ASFV) pose a significant risk to the global swine industry due to its high impact on swine health and agro-economy. Several different modes of ASFV transmission make it difficult to predict and prevent ASFV introduction to the free area and its spread in the affected area. Indirect transmission through contaminated surfaces could be one of the possible routes to introduce ASFV to the United States due to its high resistance on environmental surfaces and the frequency of international movements. However, there is limited knowledge about environmental samples for ASFV surveillance, when compared to clinical samples from infected pigs. Therefore, the aim of this study was to develop methods for better detection of ASFV DNA in the presence of four different types of organic contaminants: soil, swine feces, feed dust, and their mixture. The presence of organic contaminants negatively affected the sensitivity of ASFV DNA detection. Centrifugation and filtration were crucial for ASFV detection in environmental samples with soil and mixture, whereas filtration reduced the sensitivity of ASFV DNA detection in samples from clean surfaces and swine feces- and feed dust-contaminated surfaces. Detection of ASFV was significantly improved when sampled by the sponge stick with DNA/RNA shield when compared to the cost-effective sampling strategy, the cotton gauze with phosphate-buffered saline. These findings highlight the effect of organic contaminants and the use of the nucleic acid stabilization buffer on ASFV diagnostic performance and provide important background for ASFV preparedness.

## 1. Introduction

African swine fever (ASF) is a fatal disease in swine with up to 100% mortality. The infection of the ASF virus (ASFV) causes severe outcomes in domestic pigs and wild boars, whereas its infection in African wild suid species, such as warthogs and bushpigs, is asymptomatic. The virus is transmitted through several different routes. ASFV is the only known DNA arbovirus, and eight *Ornithodoros* species of soft tick have been demonstrated to serve as the competent vector for ASFV [1]. ASFV is mainly maintained in a sylvatic cycle between *Ornithodoros porcinus porcinus* and warthogs in sub-Saharan African [2]. The long-term persistence of ASFV in *Ornithodoros erraticus* and its transmission to pigs made it difficult to implement the eradication program in the Iberian Peninsula [3, 4]. Transmission to domestic pigs is mediated by direct contact with infected pigs and wild boars, the consumption of infected pork products or contaminated feeds, and the exposure to fomite [5]. Since the introduction of the genotype II virus into Georgia, the movement of wild boars and their interaction with domestic pigs, especially backyard pigs, play a significant role in ASFV spread from Caucasian to Eastern Europe to finally Western Europe and its persistent circulation in many European countries [6]. This is supported by the evidence that the ASF outbreak in domestic pigs is closely associated with the ASFV detection in wild boars in the same area [7]. After a gradual spread in Europe for a decade, the virus was first reported in the largest pork-producing country, China, in 2018 [8]. Subsequently, the virus quickly spread to neighboring Asian countries. In addition to the trans-continental transmission of ASFV from Africa to the Caucasian region, the ability of the virus to cross over a long distance was supported by the detection of the contemporary genotype II strain of ASFV in the Dominican Republic in 2021 [9].

Despite the greatest effects to control ASFV transmission, the virus continues to expand the geographic distribution from Africa to Europe and Asia. However, current epidemiological evidence and knowledge do not fully illustrate global ASFV transmission. For example, it still remains unclear how the virus spread rapidly among Asian countries in a short period of times since the first identification in China in 2018 and jumped intercontinentally to the Dominican Republic, potentially from Asia or Europe. The current evidence indicated that the inter-continental introduction of ASFV to the Caucasian region in 2007 could be closely related to the high stability of ASFV on pork products during personal movement and subsequent swill feeding [10]. While ASF has not been detected in mainland North America, the virus is currently present and circulating in the Dominican Republic and Haiti. Therefore, this expansive spread of ASF has been tremendously devastating, and preventing further expansion of affected areas is critical. Given that there is no safe and effective ASFV vaccine for domestic pigs and wild boars, control efforts focus on reducing transmission and spread to areas free from the disease, which can be achieved by enhanced biosecurity, early detection, and rapid response.

Several studies support the high resistance of ASFV to chemical and physical treatments and the prolonged survival, especially in raw meat, blood, organs, and processed pork products at low temperatures [11]. For example, the virus remains viable for extended periods of time, up to 204 days in raw pork, spleen, and bone marrow at 6–8°C [12]. In addition, the blood from viremic pigs remained infectious without a significant reduction of virus titer after storing at 4°C for at least 75 weeks [13]. Especially, the infectious viruses were still isolated up to 5 days in the feces and urine from ASFV-infected pigs [14]. Furthermore, the viruses still remain infectious for up to 7 days at 25°C on glass, rubber, and metal surfaces, and mathematical modeling estimated ASFV persistence 11–17 days on nonporous and 14–22 days on porous surfaces at 25°C [15]. This point suggests that indirect ASFV transmission could occur not only through the consumption of infected pork products but also through the exposure to fomites where the infectious virus is contaminated and subsequently survives. Environmental samples are useful for surveillance and evaluation of the level of contamination, which enables practitioners, swine producers, and governments to implement mitigation measures, disrupt the transmission cycle, and prevent disease transmission. For example, the testing results of the environmental samples resulted in biosecurity-focused actions, including decontamination of interior surfaces and washing of exterior trucks with high-pressure water, followed by surface disinfectants, in ASFV endemic areas [16]. In addition, environmental swab samples have been used to evaluate the level of infection and degree of contamination in ASF-affected farms [17]. Despite the importance of environmental samples in ASFV surveillance and biosecurity, there is limited knowledge on the diagnostic sensitivity of environmental samples when compared the clinical samples, such as blood and tissue samples from infected pigs or wild boars. Previously, our study evaluated ASFV detection in the environmental samples from stainless steel surfaces with no organic contaminants [18]. However, one of the greatest obstacles for the use of environmental samples for surveillance is the high level of organic contaminants, which decreases the sensitivity of the subsequent molecular analysis and increases the chance of a false negative. Therefore, in order to enhance the capacity of early detection and rapid implementation of countermeasures in ASF outbreaks, this study aimed to develop and optimize the methods for improving ASFV detection in environmental samples in the presence of organic contaminants.

## 2. Materials and Methods

### 2.1. Ethics Statement and Virus

All experiments were approved under the Kansas State University (KSU) Institutional Biosafety Committee (IBC, Protocol #1600) and performed in a biosafety level-3 laboratory at the Biosecurity Research Institute at KSU. Whole blood was collected from Georgia07-infected pigs from a separate animal study and stored at −80°C until the experiment was conducted. The virus titer was 1.36 × 10^8^ TCID_50_/mL.

### 2.2. Experiment 1

Experiment 1 aimed to determine the effect of centrifugation on ASFV detection in environmental samples in the presence of organic contaminants. One hundred microliters of blood from ASFV-infected pigs were mixed with 2.5 g of each organic contaminant and 2.5 mL of phosphate-buffered saline (PBS): (1) soil, (2) swine feces collected from finishing pigs, (3) feed dust collected from the feed mill, and (4) mixture of soil, swine feces, and feed dust. Three 10 × 10 cm stainless steel surfaces per each organic contaminant were inoculated with the mixture and were dried for 30 min. The surface was swabbed using premoistened cotton gauze (Dynarex Corporation, Orangeburg, NY, USA) with 5 mL of PBS and placed into a 50 mL conical tube. After adding 5 mL of PBS, the tube was vortexed and incubated for 5 min at room temperature. The supernatant was aliquoted into seven microtubes and centrifuged under the following conditions: no centrifugation, centrifugation for 5 min at 700 × *g* for 5, 10, and 15 min, or centrifugation at 10,000 × *g* for 5, 10, and 15 min. Centrifuge speeds were selected based on the capacity of portable and benchtop centrifuges for low and high speeds, respectively. After centrifugation, the supernatant was transferred to a new cryovial, and the equal volume of supernatant and AL lysis buffer (Qiagen, Germantown, MD, USA) was mixed and stored at –80°C until further experiment. Positive control includes 100 µL blood and 15 mL of PBS.

### 2.3. Experiment 2

In order to determine the effect of filtration on ASFV detection, three stainless steel surfaces per each organic contaminant were prepared, and the supernatant was collected, as mentioned above. After aliquoting into four microtubes, the supernatant was processed by (1) no centrifugation/filtration, (2) centrifugation at 700 × *g* for 5 min, (3) filtration through a 0.45 µm syringe filter (TPP, Trasadingen, Switzerland), and (4) centrifugation at 700 × *g* for 5 min and followed by filtration through a 0.45 µm syringe filter. Filtration was selected for potential sample processing, such as point-of-care (POC) assay. AL lysate was prepared by mixing an equal volume of the processed supernatant and AL lysis buffer and stored at –80°C until further experiment. Positive control includes 100 µL blood and 15 mL of PBS.

### 2.4. Experiment 3

This experiment compared the sensitivity of ASFV detection between two different sampling devices. Three stainless steel surfaces per each organic contaminant were prepared and swabbed using the wet cotton gauze as described above, and another set of three surfaces was sampled using the sponge stick (Cat. #SSL100, 3M, MN, USA) pre-moistened with 10 mL DNA/RNA shield (Zymo Research, Irvine, CA). The cotton gauze was placed in a 50 mL conical tube, 5 mL of PBS was added, and the tube was vortexed and incubated for 5 min. The sponge stick was placed into a plastic bag, 5 mL of PBS was added to the samples from feed dust and mix-contaminated surfaces, and then the bag was massaged and incubated for 5 min. The supernatant was transferred to the microtube and centrifuged at 700 × *g* for 5 min. The equal volume of the processed supernatant and AL lysis buffer and stored at –80°C until further experiment. Positive control includes 100 µL blood and 15 mL PBS for soil and swine feces, and 100 µL blood and 20 mL PBS for feed dust and mix.

### 2.5. Viral DNA Extraction and Quantitative PCR

Viral DNA was extracted using an automated magnetic bead-based extraction system as previously described [19]. Briefly, the AL lysate was incubated at 70°C for 10 min, and 200 µL of the lysate and 200 µL of isopropanol were added into the extraction plate. After extraction, 5 µL DNA was mixed with primers, probe, and PCR mastermix in the total volume of 20 µL. PCR was performed in duplicate using the CFX 96 PCR machine. The Cq value was converted to copy numbers/mL using the standard curve generated from serial dilutions of the known concentration of the plasmid containing the target p72 sequence. The PCR efficiency is 90%, with an *R*-value of 0.986.

### 2.6. Statistical Analysis

ASFV DNA copy number was log-transformed and analyzed in GraphPad Prism 10 (GraphPad Software, San Diego, CA, USA). Analysis of variance along with the use of a Tukey multiple comparison adjustment to control Type I error rate was used for experiments 1 and 2. For experiment 3, *t*-tests were performed to compare the ASFV DNA detection between the two treatment groups for the different sample devices. Within all statistical analyses, the positive control was excluded. Evaluation of statistical assumptions was performed using visualization of residual plots, and assumptions appeared to be reasonably met. The recovery rate was calculated as follows: DNA copy numbers of the sample/DNA copy numbers of the positive control.

## 3. Results

**Figure 1:**
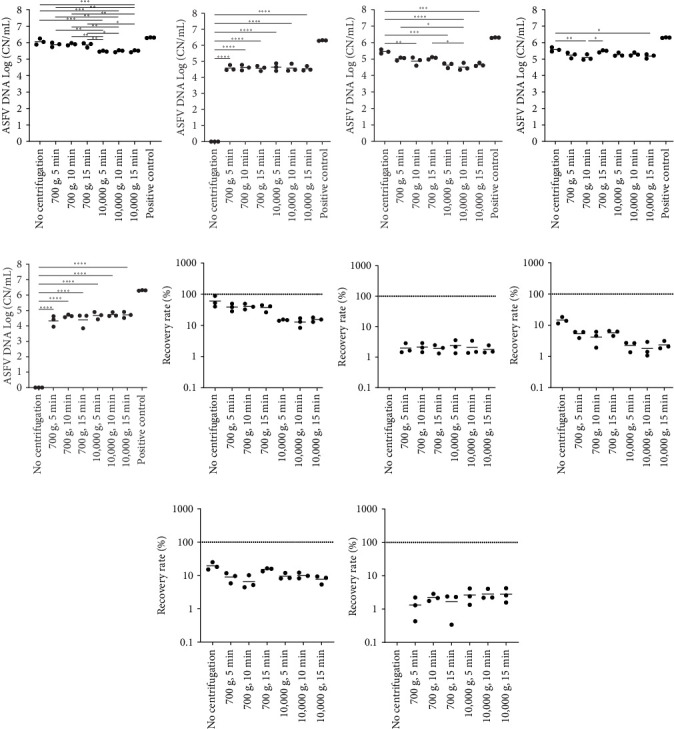
ASFV DNA detection in environmental samples. Stainless steel surfaces were inoculated with a mixture of 100 µL of ASFV-infected blood and 5 mL of PBS (A and F) or 2.5 g of each organic contaminant and 2.5 mL of PBS: soil (B and G), swine feces (C and H), feed dust (D and I), and their mixture (E and J). The surfaces were swabbed using wet cotton gauze, and the aliquots of the supernatants were centrifugated under the following conditions: no centrifugation, centrifugation for 5 min at 700 × *g* for 5, 10, and 15 min, or centrifugation at 10,000 × *g* for 5, 10, and 15 min and then subjected to quantitative PCR detecting ASFV DNA. Positive control was the same amount of blood and 15 mL of PBS. The amount of ASFV DNA (copy numbers/mL) was log-transformed for statistical analysis, and the central tendency was represented mean of log-transformed values (A–E). The recovery rate (%) was calculated by dividing the amount of ASFV DNA of the sample by that of the positive control, and the central tendency was represented mean (F–J). Statistical differences were assessed by ANOVA (*p*-value < 0.05: *⁣*^*∗*^, <0.01: *⁣*^*∗∗*^, <0.001: *⁣*^*∗∗∗*^, and <0.0001: *⁣*^*∗∗∗∗*^). (A) and (F) were adapted from “Development and optimization of sampling techniques for environmental samples from African swine fever virus-contaminated surfaces with no organic contaminants” by Kwon et al. [18], Frontiers in Veterinary Science, Figure 4A,C (https://doi.org/10.3389/fvets.2024.1425928). CC BY. ASFV, African swine fever virus; PBS, phosphate-buffered saline.

In order to determine the effect of centrifugation on ASFV DNA detection in environmental samples in the presence of organic contaminants, the environmental samples were subjected to different conditions of centrifugation. In the presence of soil in environmental samples, the samples without centrifugation were negative for ASFV DNA detection (Figure 1B,G). Viral DNA was detected in the samples containing soil after centrifugation at 700 × *g* or at 10,000 × *g*; however, the sensitivity of ASFV PCR was drastically decreased in the soil samples after centrifugation (~1% recovery rate). For swine feces samples, the highest sensitivity was identified in the samples that were not subject to centrifugation (~10% recovery rate) (Figure 1C,H). In addition, the sensitivity of ASFV DNA detection in environmental samples containing swine feces significantly decreased after centrifugation at high speed, with 1-log reduction, when compared to the samples without centrifugation. The presence of feed dust in environmental samples had a negative effect on ASFV DNA sensitivity, ~10% recovery rate (Figure 1D,I). However, the highest ASFV DNA detection was identified in the samples without centrifugation because both centrifugation at low and high speeds did not improve the sensitivity of ASFV PCR in feed dust samples. Similar to soil samples, there was no ASFV DNA detection in environmental samples from organic material mixture-contaminated surfaces without centrifugation (Figure 1E,J). Centrifugation at both high and low speeds improved ASFV DNA detection in the mixture samples, but their recovery rate still was ~1%. Generally, we did not observe the effect of time on ASFV detection in environmental samples.

**Figure 2:**
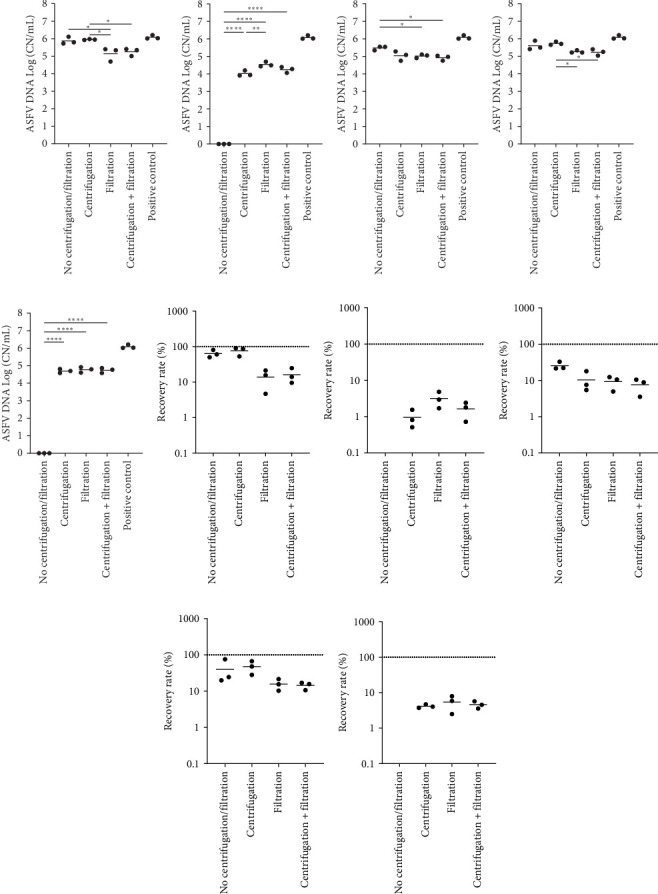
ASFV DNA detection in environmental samples. Stainless steel surfaces were inoculated with a mixture of 100 µL of ASFV-infected blood and 5 mL of PBS (A and F) or 2.5 g of each organic contaminant and 2.5 mL of PBS: soil (B and G), swine feces (C and H), feed dust (D and I), and their mixture (E and J). The surfaces were swabbed using wet cotton gauze, and the aliquots of the supernatants were processed by (1) no centrifugation/filtration, (2) centrifugation at 700 × *g* for 5 min, (3) filtration through a 0.45 µm syringe filter, and (4) centrifugation at 700 × *g* for 5 min and followed by filtration through a 0.45 µm syringe filter and then subjected to quantitative PCR detecting ASFV DNA. Positive control was the same amount of blood and 15 mL of PBS. The amount of ASFV DNA (copy numbers/mL) was log-transformed for statistical analysis, and the central tendency was represented mean of log-transformed values (A–E). The recovery rate (%) was calculated by dividing the amount of ASFV DNA of the sample by that of the positive control, and the central tendency was represented mean (F–J). Statistical differences were assessed by ANOVA (*p*-value < 0.05: *⁣*^*∗*^, <0.01: *⁣*^*∗∗*^, <0.001: *⁣*^*∗∗∗*^, and <0.0001: *⁣*^*∗∗∗∗*^). (A) and (F) were adapted from “Development and optimization of sampling techniques for environmental samples from African swine fever virus-contaminated surfaces with no organic contaminants” by Kwon et al. [18], Frontiers in Veterinary Science, Figure 4B,D (https://doi.org/10.3389/fvets.2024.1425928). CC BY. ASFV, African swine fever virus; PBS, phosphate-buffered saline.

In order to illustrate the effect of filtration of ASFV detection, four different processing techniques were tested: (1) no centrifugation/filtration, (2) centrifugation at 700 × *g*, (3) filtration, and (4) both centrifugation and filtration. Consistent to the previous findings, soil samples with no centrifugation/filtration were negative by qPCR. In contrast, centrifugation and/or filtration improved the sensitivity of ASFV PCR, and the best sensitivity was identified after filtering environmental samples (Figure 2B,G). For swine feces samples, there was no significant difference between with no centrifugation/filtration vs after centrifugation (Figure 2C,H). In contrast, the filtration reduced the detection of ASFV DNA in swine feces samples. Similarly, we found reduced sensitivity of ASFV PCR in the environmental samples that contained feed dust and were processed by filtration (Figure 2D,I). Both centrifugation and filtration enhanced the detection of ASFV DNA in environmental samples in the presence of a mixture of organic contaminants (Figure 2E,J).

**Figure 3:**
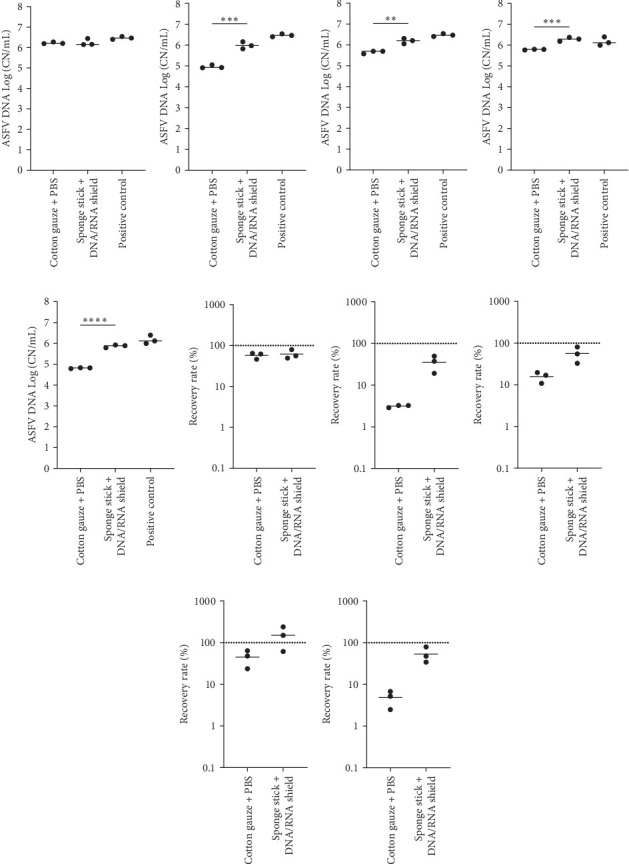
ASFV DNA detection in environmental samples. Stainless steel surfaces were inoculated with a mixture of 100 µL of ASFV-infected blood and 5 mL of PBS (A and F) or 2.5 g of each organic contaminant and 2.5 mL of PBS: soil (B and G), swine feces (C and H), feed dust (D and I), and their mixture (E and J). The surfaces were swabbed using the cotton gauze with PBS or the sponge stick with DNA/RNA shield. The supernatants were subjected to quantitative PCR detecting ASFV DNA. Positive control was the same amount of blood and 15 mL of PBS for clean, soil, and swine feces and 20 mL of PBS for feed dust and mixture. The amount of ASFV DNA (copy numbers/mL) was log-transformed for statistical analysis, and the central tendency was represented mean of log-transformed values (A–E). The recovery rate (%) was calculated by dividing the amount of ASFV DNA of the sample by that of the positive control, and the central tendency was represented mean (F–J). Statistical differences were assessed by Student *t*-test (*p*-value < 0.01: *⁣*^*∗∗*^, <0.001: *⁣*^*∗∗∗*^, and <0.0001: *⁣*^*∗∗∗∗*^). ASFV, African swine fever virus; PBS, phosphate-buffered saline.

Lastly, we compared the ASFV DNA recovery between two sampling techniques in the presence of organic contaminants. The use of cotton gauze to swab the contaminated surfaces still resulted in ASFV DNA detection in all samples. Importantly, the sensitivity of ASFV PCR was significantly improved when the surfaces were sampled by the sponge sticks pre-moistened with the commercial nucleic acid preservative (Figure 3).

## 4. Discussion

Previously, we developed and optimized the methods for detecting ASFV DNA in environmental samples from surfaces with no organic contaminants [18]. Nonetheless, there is a gap of knowledge of how to detect ASFV in environmental samples, which, in reality, are often contaminated with organic matter that could potentially inhibit and decrease the sensitivity and specificity of ASFV diagnostics because they usually contain a wide variety of PCR inhibitors that potentially interfere with the downstream. Therefore, we compared different sampling and processing techniques to better increase PCR sensitivity for environmental samples in the presence of organic contaminants. In detail, three different types of organic contaminants and their mixture were selected based on their presence in environmental samples and their relevance on ASFV transmission. Soils are present on footwear, equipment, and vehicles, all of which contribute to the spread of swine diseases across the farms and target samples for assessing biosecurity levels and preventing disease transmission. In particular, the carcasses of ASFV-infected wild boars extensively contaminate the nearby soils, and contaminated soil may be disseminated in the season of rainfalls. Second, swine feces are the common source of spreading enteric diseases, such as porcine epidemic diarrhea virus (PEDV), through fomite between swine farms. In addition, it is well documented that ASFV-infected pigs shed the virus through fecal route [14, 20, 21]. The massive international movement of feed and feed ingredients poses a great risk of the introduction of transboundary diseases in the US swine industry. Given that contaminated feed may have been responsible for the introduction of PEDV into Canada in 2014 [22], ASFV-contaminated feed and feed ingredients may serve as a reservoir of the virus for the introduction and rapid spread across the farms because the viruses were still remained infectious for at least 5 weeks in the feed ingredient stored at 4°C [22–24]. Furthermore, ASFV DNA was extensively persistent for a long period of times in feed and feed mills after experimental contamination during feed manufacturing [25]. However, there is still a gap of knowledge on the minimum infectious doses that ASFV initiates infection via natural routes, which is critical to assess the risk of fomite transmission or infection via consumption of contaminated feed and feed ingredients. Under experimental conditions, intraoropharyngeal challenge with 100 HAD_50_ of Malawi Lil-20/1 isolated resulted in one out of two pigs being infected [26]. Oro-nasal challenge with less than 10 HAU of Armenia08 strain led to the weakest animals being infected [27]. In contrast, pigs were not infected after ingesting a piece of bread spiked with 10^5.5^ HAD_50_ [28], suggesting that infectious dose would vary in different vehicles of inoculum. Further studies are required to determine the minimum infections dose in various conditions for risk assessment of different routes of infection.

Our results showed that the sensitivity of ASFV detection was greatly impaired in the presence of all types of organic contaminants when compared to samples from clean surfaces, which was consistent with the previous findings of PEDV research [25]. The degradation of ASFV DNA by nucleases and the presence of intrinsic PCR inhibitors in organic contaminants might explain the reduced ASFV detection. In particular, we observed no ASFV detection in soil- and mixture-contaminated samples without processing, implying that our magnetic bead extraction system did not eliminate PCR inhibitors that were naturally present in soil. Humic substances in soil, specifically humic acid and fulvic acid, are well-known natural PCR inhibitors and bind to (1) DNA polymerase to interfere with enzymatic processes and (2) DNA templates to inhibit amplification [29]. For these reasons, in the field of forensic science, many efforts have been implemented to remove and eliminate PCR inhibitors by optimizing pretreatment, extraction, separation, and purification steps [30]. Our results showed that these types of PCR inhibitors in ASFV-contaminated soil were partially removed by centrifugation and filtration and, subsequently, magnetic bead extraction, which resulted in a below 10% recovery rate. A previous study indicated that the ASFV BA71v strain remained infectious for up to 112 days in sand-clay soil at 4°C [31]. Another study showed that infectious ASFV was stable for up to 3 weeks in sterile sand, 2 weeks in beach sand, 1 week in yard soil, and 3 days in swamp soil [32]. For these reasons, the contaminated surfaces with soil or frequently contacted surfaces with soil are often considered the target of environmental sampling, such as footwear and vehicles. Therefore, more validated methods should be required to detect ASFV in environmental samples in the presence of soil. Swine feces contain highly variable components depending on feed type, gut flora, and environment, but generally, polysaccharides, bile salts, lipids, and urate are considered potential PCR inhibitors [33]. A variety of plant-derived compounds, such as polyphenols, polysaccharides, pectin, and xylan, can also inhibit PCR reactions in feed dust samples [34]. For these two types of samples, the highest sensitivity was found in samples with no centrifugation/filtration, implying that magnetic bead extraction was satisfactory to remove PCR inhibitors, but centrifugation and filtration did not have a positive effect on ASFV detection. In general, it is recommended that the environmental samples are centrifuged at 700 × *g* for 5 min before testing for molecular diagnostics in the lab because centrifugation is more cost-effective and less time-consuming than filtration. Moreover, unlike the present study, where the type and the volume of organic contamination were controlled in samples, we do not know the exact contents in the environmental samples in the real world; thus, it would be the key step to remove the potential PCR inhibitors through centrifugation. Furthermore, it is worth noting that all samples were positive after filtration, even though there was a significant reduction of ASFV DNA detection. Because accurate and timely detection of transboundary animal diseases is crucial to respond and control disease outbreaks, the POC tests allow rapid detection in remote and resource-limited settings. Therefore, filtration could be one of the methods for sampling processing in the field where resources for centrifugation are limited.

Another important finding of this study was that the use of the sponge stick containing the nucleic acid preservative significantly improved ASFV DNA detection in the presence of organic contaminants. Our previous study also identified the best combination of the sponge stick and the nucleic acid preservative for detecting ASFV DNA in environmental samples when small amounts of dried blood (100 µL) were swabbed on clean stainless steel [18]. In contrast, in this study, an increased volume (100 µL + 5 mL PBS) of the liquid inoculum on steel surfaces was swabbed for comparing between clean and organic contaminated conditions. The different types and volumes of the inoculum might explain why there was no significant difference in clean surfaces in this study. Several types of nucleic acid preservatives are commercially available and have been shown to improve viral detection in sample matrix containing the high level of PCR inhibitors as well as nucleases, such as stool and wastewater [35, 36]. The main purpose of nucleic acid preservatives is to stabilize the integrity of nucleic acids and prevent degradation for long-term preservation when the sample is not immediately processed or stored at −80°C. In this study, the samples were immediately processed after collection and stored at −80°C for molecular diagnostics, suggesting the positive effect of DNA/RNA shield on ASFV DNA detection in fresh samples containing organic contaminants.

## 5. Conclusion

Collectively, our study illustrated the effect of organic contaminants on ASFV detection in environmental samples and the mitigation strategies to overcome the negative effects on molecular diagnostics. We found the reduced detection of ASFV DNA in the presence of four types and of organic contaminants and their mixture, especially no detection in soil and mixture-contaminated samples. Centrifugation and filtration were crucial for ASFV detection in environmental samples with soil and mixture, whereas filtration reduced the sensitivity of ASFV DNA detection in samples from clean surfaces and swine feces- and feed dust-contaminated surfaces. Importantly, the diagnostic sensitivity was significantly recovered when organic-contaminated samples were swabbed using the sponge stick containing the commercial nucleic acid preservative. The results presented in this study have implications for ASFV surveillance and preparedness.

## Data Availability

All data are included in the article.
